# Potential Targets and Biomarkers of Radionuclide Therapy in Breast Cancer

**DOI:** 10.1002/cai2.70043

**Published:** 2026-01-17

**Authors:** Yujing Tan, Cheng Zeng, Jiani Wang, Fei Ma

**Affiliations:** ^1^ Department of Medical Oncology National Cancer Center/National Clinical Research Center for Cancer/Cancer Hospital, Chinese Academy of Medical Sciences & Peking Union Medical College Beijing China

**Keywords:** breast cancer, radionuclide therapy, targeted radionuclide therapy

## Abstract

In recent years, multidisciplinary treatment strategies have profoundly improved drug responses and survival outcomes of breast cancer (BC) patients. However, there is an urgent need for novel therapies for BC patients who are heavily treated or develop resistance to conventional treatment regimens. Radionuclide therapy (RT) and targeted radionuclide therapy (TRT) have emerged as paradigm‐shifting therapeutic approaches for BC, which enable functions of both imaging and localised treatment. They utilise radionuclides that can selectively bind to biomarkers overexpressing on BC cells, allowing precise delivery and localised tumour irradiation. Moreover, several types of radionuclides possess ‘cross‐fire’ effects that result in the eradication of neighbouring tumour cells lacking the biomarker expression. In the current review, we summarise the potential biomarkers for the development of RT and TRT that can be employed in the treatment of BC, including receptor markers of ER, PR and HER2, together with other markers of Trop2, PD‐1, EGFR, GRPR and PSMA.

## Graphical abstract

Potential biomarkers for the development of targeted radionuclide therapy that can be employed in the treatment of breast cancer have been summarised in the review. They include receptor markers of ER, PR and HER2, as well as other markers of Trop2, PD‐1, EGFR, GRPR and PSMA.

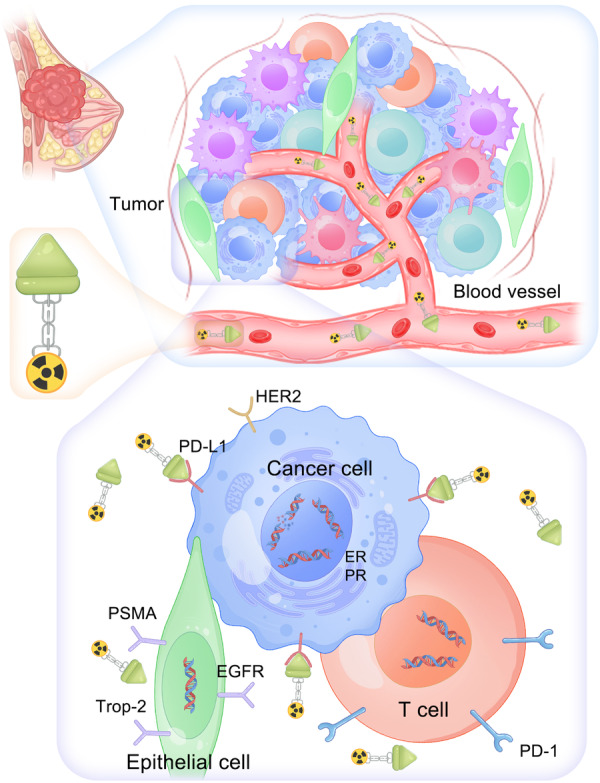

**Abbreviations**
ADCs: antibody–drug conjugates
AIs: aromatase inhibitors
BBN: bombesin
BC: breast cancer
CDK4/6i: cyclin‐dependent kinases 4/6 inhibitor
CIP: chemically induced proximity
CT: computerised tomography
CTR: covalent targeted radionuclide
DAMPs: damage‐associated molecular patterns
EGFR: epidermal growth factor receptor
ER: oestrogen receptor
ET: endocrine therapy
GRP: gastrin‐releasing peptide
GRPR: gastrin‐releasing peptide receptor
HER2: human epidermal growth factor receptor 2
HR: hormone receptor
ICIs: immune checkpoint inhibitors
mAbs: monoclonal antibodies
mBC: metastatic BC
NPs: nanoparticles
OS: overall survival
PAM pathway: phosphoinositide 3‐kinase (PI3K)/protein kinase B (AKT)/mammalian target of rapamycin (mTOR) signalling pathway
PAMAM: polyamidoamine
PARP: poly ADP‐ribose polymerase
PD‐1: programmed cell death protein 1
PET: positron emission tomography
PFS: progression‐free survival
PR: progesterone receptor
PROTAC: PROteolysis targeting chimera
PSMA: prostate‐specific membrane antigen
PTX: paclitaxel
RT: radionuclide therapy
SERD: selective oestrogen receptor downregulator
SERMs: selective oestrogen receptor modulators
SPECT: single‐photon emission computed tomography
TKIs: tyrosine kinase inhibitors
TME: tumour microenvironment
TNBC: triple‐negative breast cancer
Trop2: trophoblast cell surface antigen 2
TRT: targeted radionuclide therapy

## 1 Introduction

### 1.1 Treatment Landscape of Breast Cancer

In recent decades, the treatment landscape for breast cancer (BC) has evolved from local surgery to more personalised therapy. This evolution has been driven by the identification of particular molecular subtypes in developing treatment decisions, which are determined primarily by specific biomarkers, such as oestrogen receptor (ER), progesterone receptor (PR) and human epidermal growth factor receptor 2 (HER2) [1, 2, 3]. According to these biomarkers, BC can be categorised into at least four subtypes: hormone receptor‐positive (HR+)/HER2‐negative (HER2−), HR+/HER2‐positive (HER2+ or HER2‐enriched), HR−/HER2+ and triple‐negative breast cancer (TNBC) [4, 5].

HR+/HER2− BC represents the most prevalent molecular subtype of BC (accounting for 60%), which is generally characterised by endocrine sensitivity and favourable outcomes [6]. Endocrine therapy (ET) represents the therapeutic foundation for this particular subtype of BC, which generally contributes to lower recurrence risk and improved survival outcomes [7, 8]. In 2015, a new class of targeted drugs, the cyclin‐dependent kinase 4/6 inhibitor (CDK4/6i), was approved for use in patients with HR+/HER2− metastatic BC (mBC) because of a profound improvement of 1–1.5 years in progression‐free survival (PFS) [9, 10, 11, 12, 13]. Notably, two types of CDK4/6i, namely, ribociclib and abemaciclib, effectively prolong the overall survival (OS) of mBC patients [14, 15, 16]. Since then, the combination of CDK4/6i and ET has been developed as a new standard‐of‐care treatment regimen for patients with HR+/HER2− ABC, replacing the traditional treatment modality of single‐agent ET [17]. HER2+ breast tumours account for approximately 30% of all BC cases and are typically treated with HER2‐targeted drugs such as trastuzumab and pertuzumab [18, 19, 20]. TNBC is characterised by highly aggressive tumours and an extremely unfavourable prognosis, accounting for approximately 10% of cases [21, 22]. Cytotoxic chemotherapy remains a cornerstone treatment for this type of BC, with the addition of immune checkpoint inhibitors (ICIs) or antiangiogenic therapy further improving long‐term and short‐term outcomes [23, 24, 25, 26].

Currently, BC patients have multiple therapeutic options, including surgery, radiotherapy, chemotherapy, ET, diverse types of targeted therapy, and immunotherapy. For early‐stage BC, surgical intervention remains the primary modality offering curative intent. In patients with advanced disease, multidisciplinary treatment strategies significantly enhance drug responses and survival outcomes. Furthermore, several novel therapeutic modalities, including diverse inhibitors of the phosphoinositide 3‐kinase (PI3K)/protein kinase B (AKT)/mammalian target of rapamycin (mTOR) signalling pathway (PAM pathway), poly ADP‐ribose polymerase (PARP) inhibitors, antibody‐drug conjugates (ADCs) and proteolysis‐targeting chimaera (PROTAC) protein degraders, help to provide additional clinical benefits for patients with BC [26, 27, 28, 29, 30, 31, 32]. However, drug resistance to all these therapeutic approaches is an inevitable challenge, highlighting the exploration of innovative forms of anticancer strategies. In this context, radionuclide therapy (RT) has emerged as a potential complementary therapeutic approach, particularly for patients who have progressed on multiple lines of conventional therapy.

### 1.2 Overview of Radionuclide Therapy

RT and targeted radionuclide therapy (TRT) represent paradigm‐shifting therapeutic modalities for treating advanced or metastatic cancers, which can lead to the majority of cancer‐related deaths. Currently, RT approved by the U.S. Food and Drug Administration has become standard clinical practice for treating patients with metastatic cancer, representing a significant advancement in targeted oncologic interventions [33].

In general, RT utilises radionuclides that target particular cellular processes or accumulate in cancer cells because of their chemical properties [34]. TRT involves the administration of radiotracers designed to bind biomarkers overexpressed on cancer cells [35]. These radiotracers primarily consist of two key components: a target and a radionuclide [36]. Targets can be of diverse forms, including an antibody, a natural or synthetic ligand, or a nanobody that can be conjugated to the radionuclide via a chelator. Radionuclides that emit specific particles, which are capable of imaging and/or treatment, are employed in medical and biological research. They include α‐particles (e.g., ²²³Ra, ²¹²Bi, ²¹³Bi, ²²⁵Ac and ²²⁷Th), β⁻‐particles (e.g., ⁶⁷Cu, ⁹⁰Y, ¹³¹I, ¹⁷⁷Lu, ¹⁸⁶Re and ¹⁸⁸Re), β⁺‐particles (e.g., ¹¹C, ¹³N, ¹⁵O, ⁶⁸Ga and ¹⁸F), auger electrons (e.g., ¹¹¹In and ¹²⁵I) and γ emitters (e.g., ⁶⁷Cu, ¹⁷⁷Lu and ¹¹¹In) [33, 37].

The selection of the optimal radionuclide depends on the pharmacokinetic profile of the radiotracer, together with the type and stage of the cancer disease. For diagnostic radiotracers, it is ideal to match the radionuclide half‐life with that of the biological half‐life of corresponding agents. For example, when labelled agents are large molecules with high molecular weights that cause low clearance rates, it is appropriate to use radionuclides with longer half‐lives; for example, ^89^Zr, ^111^In and ^124^I can be selected. In contrast, shorter half‐life radionuclides, such as ^18^F, ^64^Cu and ^68^Ga, can label fragments with rapid clearance [38, 39, 40].

When a targeted therapy is being developed, the diagnostic radionuclide is expected to be replaced with a radionuclide that exhibits appropriate therapeutic effectiveness. Three types of radionuclides, namely, β^−^ emitters, α‐particles and auger electrons, are reported to have therapeutic functions [41, 42]. Among these, β^−^ emitters are considered ideal radionuclides for treating large tumours. They possess a longer path length, consequently exposing neighbouring tumour cells lacking target molecule expression to radiation. This phenomenon is known as the ‘cross‐fire’ effect [43, 44, 45]. To treat micrometastases and small clusters of cancer cells, α‐particles and auger electron emitters are advantageous because of their short range and high linear energy transfer, which results in highly cytotoxic effects [39, 40, 42].

**Figure 1:**
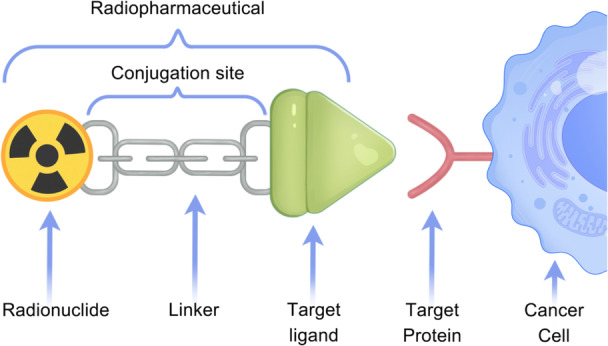
Schematic of the targeted radionuclide therapy structure. A targeted radionuclide therapy is generally composed of a therapeutic radionuclide for localised tumour irradiation (β‐particles or α‐particles), a tumour‐targeting ligand for specific interaction with the target protein on the cell surface, and a linker allowing irreversible or reversible binding of the radionuclide and target ligand.

From the perspective of drug development, a first‐generation radiopharmaceutical consists of a dual‐function conjugate linking a radionuclide (β‐particles or α‐particles) with a target on specific markers of tumour cells. In this approach, a radionuclide‐labelled module that can be selectively delivered to cancer cells is used, allowing for localised irradiation of tumour tissues [33, 34]. However, insufficient tumour retention and rapid blood clearance compromise the antitumour efficacy of such first‐generation treatment modalities because of the reversible interaction of most ligands [37, 46]. Second‐generation radiopharmaceuticals, also referred to as TRT agents, represent a significant evolution from conventional designs. TRT agents are engineered as trifunctional conjugates, comprising a therapeutic radionuclide for localised tumour irradiation, a tumour‐targeting ligand for precise delivery, and a linker that enables specific binding to cell‐surface targets [35]. Additionally, an improved structure, called a covalent targeted radionuclide (CTR), further enhanced the duration of tumour retention and the antitumour efficacy of TRT agents [47, 48]. CTRs introduced an improvement at the site of conjugation, resulting in the application of a covalent warhead allowing irreversible binding to the targeting molecule, which significantly prolonged the duration of therapeutic radionuclide efficacy [49]. A schematic of the TRT structure is illustrated in Figure 1.

**Figure 2:**
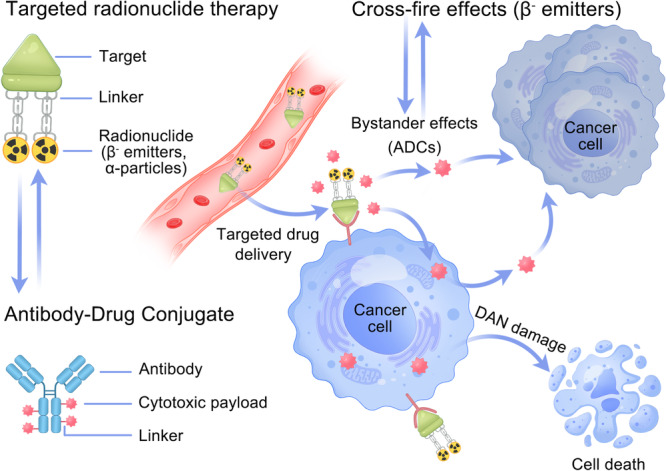
Schematic diagram of the tumour‐killing process of targeted radionuclide therapy. As a trifunctional conjugate, targeted radionuclide therapy (TRT) agents comprise a target, a linker and a radionuclide and are analogous to an ADC. Similarly, TRT agents have a target ligand to achieve high‐affinity binding to tumour‐associated markers, allowing precise radiation of cancer cells overexpressing specific markers. Moreover, TRT agents engineered with β^−^ particles exhibit ‘cross‐fire effects’ that eliminate neighbouring tumour cells lacking target marker expression.

To some extent, second‐generation TRT agents share a similar structure with ADCs, embodying a therapeutic strategy termed the ‘magic bullet’ [50, 51, 52]. Analogous to ADCs, TRT agents contain a high‐affinity ligand to target tumour‐associated markers, enabling precise delivery and localised elimination in tumour cells [35, 51]. Both of them display ‘bystander effects’, allowing for tumour‐killing effects on neighbouring tumour cells without target molecule expression (Figure 2). In contrast, ADCs require cellular internalisation mediated by cell‐surface receptors to release the cytotoxic payload through linker cleavage from lysosomal degradation [51]. In contrast, TRT agents carry β‐particles or α‐particles to exert radiation on the cell surface at the tumour site without the need for endocytosis, which can directly kill tumour cells [36].

### 1.3 Radionuclide Therapy, Targeted Radionuclide Therapy, and Breast Cancer

The success of RT and TRT is achieved by excellent tumour targeting, profound antitumour efficacy and manageable toxicity. BC constitutes highly heterogeneous tumours [53, 54, 55], necessitating the development of individualised therapies. The incorporation of radiopharmaceutical agents into the treatment of BC is reasonable. First, RT or TRT agents can bind to specific cancer‐expressing markers (such as HER2) with acceptable affinity and can deliver β‐ or α‐particles for localised irradiation, which has demonstrated a potent ability for tumour targeting and tumour regression [18, 38, 56, 57]. Second, systemic toxicity from such targeted therapy, especially TRT, is theoretically controllable. The covalent warhead of TRT agents presents a critical structure for minimising systemic toxicity on the premise of ensuring effectiveness [49, 58]. It can noncovalently bind to the tumour‐associated target and then irreversibly covalently bind via proximity, thus enhancing tumour retention and limiting the clearance rate. Owing to the relative bioorthogonality of these covalent warheads, other free covalent radioligands that do not bind to the target would experience rapid blood clearance without off‐target ligation, minimising the risk of toxicity [48, 59]. In addition to the short half‐lives of radionuclides of 3–10 days, RT and TRT agents feature good safety profiles [46]. Third, the unique characteristics of radiopharmaceutical agents in contrast to those of traditional systemic chemotherapy agents confer an inherent theranostic property [38, 60, 61]. By leveraging the intrinsic properties of radioactive particles, RT enables real‐time assessment of tumour responses or tumour heterogeneity by a simple non‐invasive imaging scan that can include the whole body in the field of view. This can be achieved by positron emission tomography (PET) or single‐photon emission computed tomography (SPECT)–computerised tomography (CT) [61]. In this way, RT permits concurrent diagnosis and treatment.

In summary, RT and TRT are promising avenues for the management of BC, given their well‐documented tumour targeting abilities, sufficient tumour retention, favourable safety profiles and inherent diagnostic functions.

## 2 Potential Targets for Radionuclide Therapy and Targeted Radionuclide Therapy in Breast Cancer

**Figure 3:**
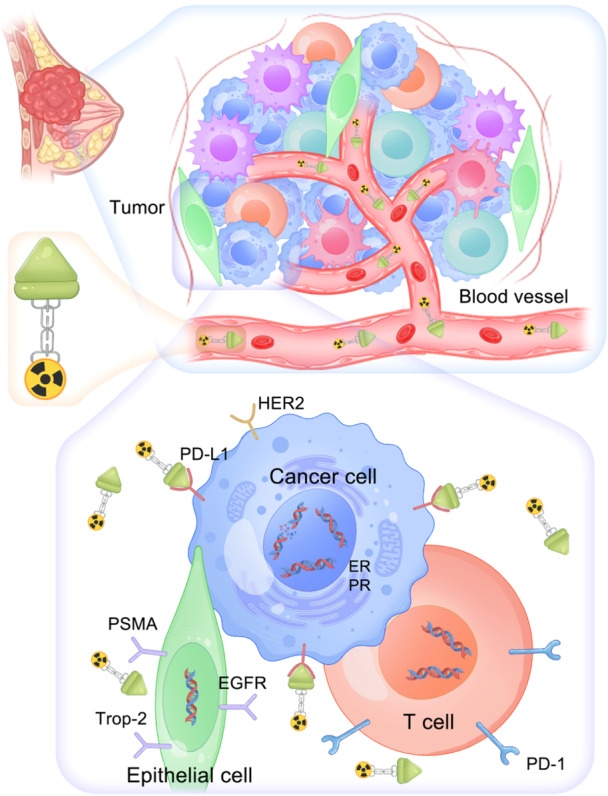
Potential biomarkers for the development of TRT agents in the field of breast cancer, including biomarkers that are expressed mainly on cancer cells (HER2, ER, PR and PD‐L1), epithelial cells (Trop2, EGFR and PSMA) and T cells (PD‐1).

The identification and targeting of molecular receptors, such as HER2, ER and PR, have transformed the therapeutic landscape of BC, leading to more personalised and effective treatment strategies [62]. A number of emerging biomarkers have further increased the number of therapeutic options available for patients with BC [63, 64, 65]. Potential biomarkers for use in TRT are illustrated in Figure 3. In theory, markers that can function as druggable targets can be integrated into RT and TRT. The integration of advanced imaging techniques and biomarker assessments will play a critical role in optimising treatment strategies and ensuring that patients receive the most appropriate therapies on the basis of their unique tumour profiles. A wide array of TRT and RT agents for the treatment of breast tumours is summarised in Table 1.

**Table 1:** Options of RT and TRT agents in the field of breast cancer.

Target	Radionuclide	Particles	Formula	Settings	Application
HER2	^188^Re	β/γ	^188^Re‐SOCTA‐trastuzumab	Preclinical	Imaging
	^68^Ga	β	^68^Ga‐NODAGA‐SNA004‐GSC	Preclinical	Imaging
	^177^Lu	β	^177^Lu‐CHX‐A‐DTPA‐trastuzumab	Preclinical	Therapy
	^177^Lu	β	^177^Lu‐CHX‐A‐DTPA‐Fab(2)‐trastuzumab	Preclinical	Therapy
	^131^I	β	^131^I‐NM‐02	Preclinical	Imaging and therapy
	^111^In	Auger/γ	^111^In‐BnDTPA‐trastuzumab‐NLS	Clinical	Imaging
	^131^I	β	^131^I‐GMIB‐anti‐HER2‐VHH1	Clinical (Phase I, recruiting)	Imaging and therapy
	^68^Ga	β	^68^Ga‐ABY‐025	Clinical	Imaging
	^68^Ga	β	^68^Ga‐NODAGA‐ADAPT6	Clinical	Imaging
	^177^Lu	β	^177^Lu‐trastuzumab	Clinical	Imaging and therapy
	^89^Zr	β	^89^Zr‐trastuzumab	Clinical	Imaging and therapy
Trop2	^99m^Tc	β	[^99m^Tc]Tc‐MY6349	Clinical	Imaging
EGFR	^177^Lu	β	^177^Lu‐T‐AuNP	Preclinical	Imaging and therapy
EGFR and GRPR	^177^Lu	β	^177^Lu‐BN‐PLGA‐(paclitaxel)	Preclinical	Imaging and therapy
GRPR	^99m^Tc	^99m^Tc	[^99m^Tc]Tc‐DB 15(N4‐AMA‐DGA‐[DPhe6‐Sar11‐Leu13‐NHEt] BBN (6–14)	Preclinical	Imaging
	^64^Cu	β	[^64^Cu]Cu‐SAR‐BBN	Clinical	Imaging and therapy
	^99m^Tc	^99m^Tc	^99m^Tc‐RP527	Clinical	Imaging and therapy
	^212^Pb	α	[^212^Pb]Pb‐DOTAM‐GRPR1	Clinical (Phase I, recruiting)	Imaging and therapy
PSMA	^177^Lu	β	^177^Lu‐labelled PSMA‐617	Preclinical	Imaging and therapy
	^177^Lu	β	[^177^Lu]Lu‐PSMA‐I&T	Preclinical	Imaging and therapy
	^68^Ga	β	[^68^Ga]‐PSMA‐11	Preclinical and clinical	Imaging and therapy
	^177^Lu	β	[^177^Lu]Lu‐PSMA	Clinical	Imaging and therapy

Abbreviations: EGFR, epidermal growth factor receptor; GRPR, gastrin‐releasing peptide receptor; PSMA, prostate‐specific membrane antigen; TRT, targeted radionuclide therapy.

### 2.1 Receptor‐Based Targets: HER2, ER and PR

HER2, ER and PR are pivotal biomarkers for BC cells and play predominant roles in classifying molecular subtypes, guiding treatment decisions and performing prognostic assessments. Targeting these three receptors has promoted the discovery of diverse drugs for the treatment of BC, generally shaping the therapeutic landscape of BC.

#### 2.1.1 HER2 as a Target

HER2, a transmembrane protein with tyrosine kinase activity, is a member of the HER family [66]. The protein structure comprises three distinct domains: an extracellular ligand‐binding domain, a single‐pass transmembrane domain and an intracellular protein tyrosine kinase domain. The intracellular kinase domain represents the pivotal functional region responsible for HER2 activation [67, 68]. HER2 is overexpressed in approximately 20%–30% of BC cells and is frequently associated with aggressive behaviours and a poor prognosis [69]. It plays a critical role in tumourigenesis and cancer progression, establishing it as a prominent and promising therapeutic target for tumour‐specific therapies [20, 70, 71]. A diverse array of HER2‐directed agents—including monoclonal antibodies (mAbs), tyrosine kinase inhibitors (TKIs) and ADCs—have been developed, significantly improving survival outcomes for patients with HER2+ BC in both early and advanced stages [72, 73, 74, 75, 76]. Notably, a type of ADC called trastuzumab deruxtecan (T‐DXd, DS‐8201) has the potential to redefine the first‐line treatment paradigm for patients with HER2+ mBC. Recent data revealed that the T‐DXd plus pertuzumab regimen contributed to an improved median PFS of 40.7 months compared with 26.9 months for the trastuzumab plus pertuzumab plus taxane regimen [77].

Although HER2+ BC patients have multiple therapeutic options, including chemotherapy, mAbs, TKIs and ADCs, the development of drug resistance remains an inevitable challenge. Moreover, patients with HER2+ BC exhibit a heightened propensity for brain metastases, which has been reported to range from 35% to 50% [78, 79]. In general, brain metastasis is associated with worse survival outcomes and limited treatment options. Following brain metastasis, the median OS ranges from 16 to 19 months for patients with HR+/HER2+ mBC and 11–13 months for those with HR–/HER2+ mBC [80]. The blood–brain barrier (BBB) impedes the penetration of most therapeutic agents, preventing them from reaching effective concentrations within the brain parenchyma. Consequently, local therapies such as surgical resection, whole‐brain radiation therapy and stereotactic radiosurgery remain the primary treatment strategies for established brain metastases [81, 82, 83]. Compared with mAbs, ADCs and TKIs, TRT agents typically possess a lower molecular weight, which facilitates greater drug concentrations. In addition, TRT agents can achieve potent antitumour effects at the surface of tumour cells expressing specific markers through localised radiation, potentially overcoming the limitations of conventional systemic drugs.

Currently, RT and TRT have been extensively explored in the treatment of HER2+ BC. HER2 represents the most well‐established target for both RT and TRT agents within the landscape of BC therapeutics. One of the most common procedures is to label trastuzumab with radionuclides, which enables imaging or antitumour activity depending on the variety of the radionuclide. In HER2+ SK‐BR‐3 and MDA‐MB‐453 human BC cells in vitro, trastuzumab labelled with ^177^Lu showed a cytotoxic effect on tumour cell proliferation [84]. With respect to in vivo models, a radioimmunoagent, ^188^Re‐SOCTA‐trastuzumab, which is formed by ^188^Re‐trastuzumab via the bifunctional ligand succinimidyl3,6‐diaza‐5‐oxo‐3‐[2‐((triphenylmethyl)thio)ethyl]‐8‐[(triphenylmethyl)thio]octanoate (SOCTA), strongly accumulated in tumour lesions in mice bearing xenografted HER2‐overexpressing BC tumours [85]. ^225^Ac‐labelled trastuzumab has been demonstrated to inhibit SUM225 ductal carcinoma in situ in mice after intraductal injection [86]. These preclinical data reveal the specific affinity and inhibitory effects of HER2‐targeting radionuclides in HER2+ BC.

In a real‐world setting, trastuzumab labelled with radionuclides has the potential to act as a practical tool for mapping tumour heterogeneity in lesions with diverse HER2 expression levels. ^177^Lu‐labelled trastuzumab administered to patients with HER2+ BC localised in primary and metastatic tumours showed high contrast on imaging by SPECT/CT, indicating its potential for the diagnosis of HER2‐positive BC [87, 88]. In a study including 60 patients with HER2+ mBC, ^89^Zr‐trastuzumab was used to successfully identify patients who could benefit from the HER2‐targeting ADC trastuzumab emtansine (T‐DM1) on the basis of HER2 expression [89].

In another prospective pilot study, HER2‐targeted PET imaging with [^68^Ga]Ga‐ABY‐025 was performed in 10 patients with HER2‐low mBC to assess disease heterogeneity. Eighty percent of the HER2‐low lesions were detected by [^68^Ga]Ga‐ABY‐025 PET, which was further confirmed by immunohistochemistry or in situ hybridisation. Substantial intra‐ and interindividual heterogeneity was observed in [^68^Ga]Ga‐ABY‐025‐avid lesions. This study demonstrated the feasibility of visualising HER2‐low disease and subsequently stratifying patients who can respond to HER2‐targeted drugs [90]. The therapeutic efficacy and toxicity profiles of these agents are expected to be investigated in future clinical trials.

Other novel anti‐HER2 targeted radionuclide agents have shown potential for imaging and therapeutic effectiveness. ^131^I‐NM‐02, an anti‐HER2 RT agent labelled with a β‐particle emitter, was observed to have apparent tumour uptake, rapid blood clearance and a favourable biodistribution in HER2+ tumour‐bearing mice. As expected, it could significantly inhibit tumour growth and extend the life of these mice with good biocompatibility [91]. In humans, a phase I clinical trial was performed to evaluate the toxicity, distribution and uptake of a targeted radionuclide theranostic agent directed at HER2+ BC, ^131^I‐GMIB‐anti‐HER2‐VHH1 [92]. The results demonstrated a manageable safety profile and profound uptake in targeted lesions in HER2+ mBC patients who developed resistance to trastuzumab, pertuzumab or T‐DM1 [92]. These results indicate that HER2 could serve as a promising druggable target for the discovery of RT and TRT agents.

HER2 remains an exceptionally promising therapeutic target for TRT development, given its distinct pathophysiological characteristics and high target specificity. Although HER2‐targeted RT and TRT approaches have yielded promising results in preclinical and clinical studies, there is a long way to go before they can be applied in clinical practice in patients with HER2+ BC. The full potential of RT and TRT in this setting awaits confirmation through additional high‐quality clinical evidence.

#### 2.1.2 ER and PR as Potential Targets

ER and PR serve as critical biomarkers in HR+ BC patients. The targeting of ER and PR has prompted the development of a series of ET agents for the treatment of HR+ BC [93]. ER is considered to be a nuclear transcription factor that can bind to oestrogen or anti‐oestrogen pharmaceuticals to hinder the replication and proliferation of BC cells [94, 95]. The first type of ER antagonist, tamoxifen (TAM), was the earliest targeted drug in the field of BC therapy [96]. TAM competes with E2 to bind ER and form the TAM complex in the cytoplasm, which affects transcriptional activity in the nucleus. With continued drug discovery, distinct new types of ET agents with greater ER selectivity have been introduced, including other types of selective oestrogen receptor downregulators (SERDs) such as fulvestrant, selective oestrogen receptor modulators (SERMs) and aromatase inhibitors (AIs) [8, 97]. Since the advent of CDK4/6i, the use of SERMs and AIs has become the standard practice for treating the HR+/HER2− BC subtype, contributing to a favourable prognosis [98].

CDK4/6i‐based therapy has transformed the therapeutic landscape for HR+/HER2− mBC, establishing itself as the standard first‐line therapy in combination with ET [15, 17, 99]. However, the optimal strategy following progression on first‐line CDK4/6i plus ET remains a critical unmet clinical need [100]. For patients who develop drug resistance to CDK4/6i and those who are heavily pretreated after multiple lines of systemic therapy, novel therapeutic strategies are highlighted. Chemically induced proximity (CIP) technologies have been explored for their ability to treat ER+ BC. CIP agents generally function via a ternary complex similar in structure to ADCs [101, 102]. This structure consists of a ligand specific for the target protein overexpressed in tumours, a ligand binding an effector protein essential for cell viability, and a linker connecting these two ligands. PROTAC can lead to robust ER degradation and subsequent inhibition of breast tumour cells in preclinical settings [103, 104]. Recently, vepdegestrant, a type of PROTAC ER degrader, has been reported to have potent antitumour efficacy and manageable toxicity in patients with locally advanced or metastatic ER+/HER2− BC who have progressed to CDK4/6i‐based therapy [105].

Based on its expression and prognostic role in BC, ER has potential as a promising target for RT and TRT, offering a tailored therapeutic avenue for those who are heavily treated. In addition, cross‐talk between oestrogen and growth factor signalling cascades has the potential to suppress the effects of radiation. In contrast, precise inhibition of ER activity can potentially increase tumour cell sensitivity to radiotherapy, suggesting that ER can be utilised in the development of RT or TRT agents [106, 107].

Although studies on RT and TRT focusing on ER and PR as targets are scarce, ER and PR are logically likely to function as targets for RT agents.

### 2.2 Trop2

Trophoblast cell surface antigen 2 (Trop2) is a member of the GA733 gene family that is expressed primarily in epithelial cells and functions in regulating intracellular calcium levels [108, 109, 110]. Structurally, the Trop2 protein is a type I transmembrane glycoprotein that features an extracellular domain, a transmembrane region and a cytoplasmic tail [110]. These properties enable Trop2‐specific ADCs to be efficiently internalised into cancer cells and trafficked to lysosomes, where they release their cytotoxic payloads [51, 109]. Crucially, many of these payloads exhibit enhanced cell membrane permeability, facilitating a potent bystander effect that can kill neighbouring tumour cells regardless of their Trop2 expression level. For example, SG and datopotamab deruxtecan (Dato‐DXd) have been shown to elicit this bystander effect, displaying promising clinical efficacy across a spectrum of solid tumours in clinical trials [111, 112, 113, 114].

In the phase III ASCENT trial, SG significantly prolonged PFS and OS in patients with relapsed or refractory metastatic TNBC (mTNBC) compared with the physician's choice of chemotherapy involving eribulin, vinorelbine, capecitabine or gemcitabine [115]. This success led to the regulatory approval of SG, establishing it as the first globally approved Trop2‐targeted ADC for the treatment of mTNBC. Another phase III TROPiCS‐02 trial evaluated the efficacy and safety of SG in patients with HR+/HER2− mBC who had received ET and ≥ 2 lines of systemic therapy in the context of metastasis [113, 116]. Similarly, the results showed that SG resulted in significantly improved PFS and OS compared with the physician's therapeutic choice [113]. Collectively, these results indicate that SG offers superior improvements in PFS and OS over chemotherapy regimens from physicians, not only for patients with relapsed or refractory mTNBC who have limited treatment options and poor prognoses but also for heavily pretreated patients with ET‐resistant HR+/HER2− BC.

Trop2 is markedly overexpressed in approximately 80% of BC lesions, with 88% of these being TNBC [117, 118, 119, 120, 121]. Trop2 expression is coregulated by epigenetic modifications and epithelial–mesenchymal transition transcription factors. The upregulation of Trop2 promotes tumour growth, proliferation and invasion by modulating key intracellular signalling pathways [110, 118]. This distinct expression profile and mechanistic involvement render it an attractive therapeutic target in BC. The success of Trop2‐specific ADCs for BC indicates that TRT agents targeting Trop2 could potentially be used for significant biomarker detection. In a pilot clinical study, a Trop2‐specific radionuclide, [^99m^Tc]Tc‐MY6349, was shown to have acceptable safety and feasibility for detecting Trop2 expression in BC [122]. Under SPECT/CT imaging, [^99m^Tc]Tc‐MY6349 exhibited greater tumour uptake in breast tumours that highly expressed Trop2 than in normal organs, highlighting the heterogeneity of Trop2 expression in BC tissues. Another retrospective study investigated the safety profile of SG plus radiation therapy in 13 BC patients and reported limited radiation‐induced toxicity and treatment interruption [123]. These findings suggest the profound tolerance of the combination of Trop2‐targeted cell death and radiation in the field of treating BC, providing a rationale for introducing Trop2‐specific RT and TRT agents.

Although Trop2‐specific radionuclides are in the early stage of exploration for use in the diagnosis of TNBC, they should be developed as radiotherapeutic targets for RT and TRT, given these favourable features, which may provide therapeutic advancements in TNBC patients with limited therapeutic alternatives.

### 2.3 Immune Checkpoints (PD‐1/PD‐L1)

The success of immunotherapy depends on T‐cell infiltration into the tumour microenvironment (TME) and the activation of effector T lymphocytes. However, tumour cells develop diverse mechanisms to impair antitumour immunity, resulting in the failure of T‐cell activation. A promising strategy to overcome this challenge involves combining immunotherapies with other treatment modalities designed to augment T‐cell infiltration and/or function within the TME [124]. BC has long been considered to have a ‘cold’ TME characterised by a low level of immune cell infiltration or the presence of stromal cell infiltration [24]. Given that BC lesions are tumours with a ‘cold’ TME, the development of immunotherapy for BC is challenging and lags far behind that for other solid tumours. In recent years, a relatively high percentage of TNBC tumours have been classified as having a ‘hot’ TME characterised by high levels of adaptive and active innate immune cells, suggesting a profound anticancer immune response [125]. Indeed, immunotherapy has achieved clinical benefits in populations of TNBC patients, such as in the KEYNOTE‐522, I‐SPY2 and IMpassion‐031 studies [126, 127, 128, 129, 130]. In the KEYNOTE‐522 study, the addition of the programmed cell death protein 1 (PD‐1) inhibitor pembrolizumab to the TP‐AC regimen as a neoadjuvant therapy effectively improved the pathological clinical response (pCR) rate (short‐term efficacy), event‐free survival and OS (long‐term efficacy) in patients with early‐stage TNBC [25, 131]. In another study, the programmed cell death ligand 1 (PD‐L1) inhibitor atezolizumab plus a chemotherapy regimen significantly improved the pCR rate in TNBC patients with positive PD‐L1 expression [128]. These findings suggest that immune checkpoints, such as PD‐1 or PD‐L1, can serve as druggable targets in the treatment of BC, especially TNBC.

Numerous studies have indicated that TRT can induce tumour‐specific immune responses or reprogramme the TME, thereby enhancing immune cell recognition and immune‐mediated tumour cell death [132, 133, 134, 135, 136]. Specifically, TRT plays a regulatory role through enhancing CD8+ T‐cell infiltration, checkpoint molecule expression on CD8+ T cells, and PD‐L1 expression on myeloid cells. This can explain why most radioligands target PD‐L1 expressed on tumour or myeloid cells but do not directly irradiate PD‐1 on T cells. Furthermore, localised irradiation can elicit a systemic antitumour effect, known as the ‘cross‐fire’ effect, which manifests in non‐irradiated tumours or metastases [45]. This effect is mediated through multiple mechanisms, including the release of abundant endogenous damage‐associated molecular patterns (DAMPs) from apoptotic tumour cells via a process termed immunogenic cell death. These DAMPs subsequently activate dendritic cells, promoting enhanced tumour antigen presentation, stimulating cytotoxic T lymphocytes and triggering the release of cytokines and chemokines that collectively potentiate an antitumour immune response [137, 138]. In an in vitro study involving the MDA‐MB‐231 and ZR75‐1 cell lines, a type of RT called ^223^Ra‐dichloride was reported to potentiate the T‐cell‐mediated elimination of BC cells following radiation, as well as the expression of calreticulin and MHC‐I molecules that facilitate and support the initiation of anticancer immunity [139]. These findings provide a rationale for combination strategies involving ICIs and radiotherapy‐based treatments, such as TRT. However, several points warrant further exploration. First, the management of combinations of TRT and ICIs should be optimised, involving the sequencing of drug administration, synergistic potential, safety profiles and context‐specific limitations. Second, the identification of potential immunological biomarkers can provide additional monitoring of therapeutic effects, such as STING pathway activation, type‐I interferon response or T‐cell infiltration [140].

In summary, targeting immune checkpoints in the development of RT and TRT agents is a relevant strategy that could enhance the antitumour efficacy of both RT and anti‐PD‐L1 therapy.

### 2.4 Other Emerging Targets

#### 2.4.1 EGFR

Epidermal growth factor receptor (EGFR, HER1) is located in the p12–p13 region of human chromosome 7 and has a molecular weight of 170 kDa [141, 142]. The protein serves as a transmembrane glycoprotein and is a member of the HER family (which contains HER1, HER2, HER3 and HER4) [141]. Multiple studies have shown that the expression of EGFR is upregulated in BC tissues compared with normal breast tissues, and among all BC subtypes, TNBC has the highest expression of EGFR [143, 144]. The EGFR signalling pathway has been reported to participate in therapeutic resistance, maintenance of stem‐like tumour cells and immunological regulation in BC [145, 146, 147]. BC patients exhibiting EGFR overexpression typically present with lower tumour differentiation grades, higher clinical stages and higher rates of lymph node metastasis [148, 149]. These findings suggest a correlation between EGFR overexpression and aggressive behaviours in BC.

An EGFR‐targeted TRT agent called ^177^Lu‐T‐AuNP is made up of the emitter ^177^Lu, an inhibitor that selectively binds to EGFR (panitumumab), and polyethylene glycol (PEG) chains derivatised with 1,4,7,10‐tetraazacyclododecane‐1,4,7,10‐tetraacetic acid (DOTA) chelators. ^177^Lu‐T‐AuNP was effective for targeting the EGFR‐overexpressing MDA‐MB‐468 cell line, with a cross‐fire effect on other neighbouring BC cells [150, 151]. In xenograft mouse models bearing EGFR+ TNBC, ^177^Lu‐T‐AuNP exhibited high tumour uptake and subsequent tumour inhibition, thereby prolonging the survival time of the experimental mice [152]. A ^177^Lu‐labelled RT agent was developed using a trastuzumab Fab linked through a PEG24 spacer to epidermal growth factor and showed specificity for both HER2 and EGFR. The dual‐targeted agent was reported to be effective for HER2‐ and EGFR‐expressing BC cells in vitro and in athymic mice in vivo [153].

EGFR represents a potential target for RT and TRT in the treatment of BC, particularly for TNBC subtypes. However, no clinical data have been reported for EGFR‐targeted RT and TRT, and more research is needed.

#### 2.4.2 GRPR

Gastrin‐releasing peptide receptor (GRPR), a bombesin (BBN) receptor and G‐protein coupled receptor subtype, is overexpressed in several cancer types [154, 155, 156]. Gastrin‐releasing peptide (GRP) binds to GRPR with high affinity and induces a variety of pharmacological and biological activities. These activities include the release of hormones from gastrointestinal and endocrine organs, central regulation of temperature and circadian rhythms, smooth muscle contraction, regulation of the immune response, and mitotic activity in human tumours [157, 158, 159]. With respect to breast tumours, 70% of Luminal A and B subtypes exhibit high GRPR expression [160]. In addition, there is a profound positive association between high ER and GRPR expression at both the mRNA and protein levels [161, 162, 163]. Hence, GRPR represents a highly relevant theranostic target in BC, particularly for ER+ subtypes.

Multiple GRPR‐targeted agonists and antagonists have been applied for PET/CT imaging in BC, including with the radionuclides ^64^Cu, ^68^Ga, ^177^Lu and ^99m^Tc [164, 165, 166]. For instance, in the T47D cell line in vivo, the radiotracer [^99m^Tc]Tc‐DB 15 (N4‐AMA‐DGA‐[DPhe6‐Sar11‐Leu13‐NHEt] BBN (6–14)) demonstrated excellent metabolic stability and enhanced GRPR‐specific uptake in breast tumours [167]. Moreover, the heterodimeric radiopeptide [^177^Lu]Lu‐DOTA‐DN(PTX)‐BN showed high, specific and rapid tumour uptake with a long retention time in xenografted T47D tumours in mice, with high‐contrast visualisation on PET/CT [168]. In some preclinical settings, GRPR‐targeted RT and TRT showed feasibility in terms of both imaging and anticancer efficacy. A targeted controlled‐release nanodrug, [^177^Lu]Lu‐BN‐PLGA(PTX), was functionalised with the chemotherapeutic drug paclitaxel (PTX) and a radionuclide, enabling concomitant imaging, radiotherapy and chemotherapy of BC. In mice bearing subcutaneous MDA‐MB‐231 xenografts, the nanodrug not only allowed the visualisation of breast tumours and metastatic lung lesions but also demonstrated the greatest tumour inhibition, with a reduction in tumour volume and SUV values compared with those in the control group [164].

Additionally, a series of GRPR‐targeted radiotracers have been used in imaging BC in patients. The radiotracer [^99m^Tc]Tc‐RP527, containing a tripeptide formed by dimethylated glycine, L‐serine and acetamidomethyl L‐cysteine (dmGly‐L‐Ser‐Acm‐L‐Cys), and the N3S chelator for radiolabelling with technetium‐99m, showed specific tumour uptake and imaging features in primary tumour tissues and metastatic lymph nodes in patients with BC [167]. These tumour tissues highly expressed GRPR, as determined by immunochemical analysis. In a phase I/II study, researchers investigated the safety and feasibility of [^64^Cu]Cu‐SAR‐BBN PET/CT in restaging patients with HR+/HER2− mBC [166]. High uptake and avidity of [^64^Cu]Cu‐SAR‐BBN were observed in ER+/PR+/HER2− BC patients, with a manageable profile. Recently, a new phase I clinical trial has been initiated to explore the safety and anticancer efficacy profiles of [^212^Pb]Pb‐DOTAM‐GRPR1 in BC patients with tumours expressing GRPR at various levels, as well as other types of cancer (NCT05283330). [^212^Pb]Pb‐DOTAM‐GRPR1 is a pharmaceutical radioimmunoconjugate constructed from an α‐particle, a metal chelator, DOTAM (1,4,7,10‐tetrakis(carbamoylmethyl)−1,4,7,10‐tetraazacyclododecane) and a GRPR‐targeted antagonist. However, to date, clinical data concerning BC have not been reported.

Currently, radiopharmaceuticals targeting GRPR are primarily applied for PET/CT imaging, whereas antitumour treatment has been attempted only in preclinical cell and mouse models. However, it is reasonable to expect GRPR to serve as a therapeutic target in the development of RT and TRT agents for the treatment of BC.

#### 2.4.3 PSMA

Prostate‐specific membrane antigen (PSMA), encoded by the FOLH1 gene, is a type II transmembrane glycoprotein consisting of an extracellular domain, a transmembrane domain and an intracellular domain [169, 170]. The extracellular domain is a major binding site for various antibodies or peptides, making it a proper biomarker for targeted therapy [171, 172]. PSMA was initially recognised for its frequent overexpression in prostate cancer [170]. Since then, it has been found to be highly expressed in other types of solid cancer, including BC. Among all BC subtypes, TNBC exhibits higher levels of PSMA expression, which are generally correlated with worse survival outcomes [173, 174]. Notably, PSMA overexpression is observed in the vasculature but not in TNBC cells themselves. Mechanistically, TNBC cells facilitate vessel formation, which is accompanied by the upregulation of PSMA expression on endothelial cells, increasing the aggressive behaviours of tumour cells [175, 176].

Currently, there are multiple types of PSMA‐targeting radiopharmaceuticals for use in the imaging and treatment of TNBC. Specific accumulation of [^68^Ga]‐PSMA‐11 was detected in the TNBC cell line MDA‐MB231 by PET imaging and was not detected in the ER‐expressing BC cell line MCF‐7 [171]. ^177^Lu‐labelled PSMA‐617 potently impaired the proliferation and angiogenic potential of HUVECs, which play crucial roles in angiogenesis in TNBC [171]. Similarly, another preclinical study assessed the therapeutic efficacy of [^177^Lu] Lu‐PSMA‐I&T endogenous radiotherapy in an orthotopic mouse model of TNBC [176]. [^177^Lu] Lu‐PSMA‐I&T was administered to mice in single‐ and fractionated‐dose approaches, while mice in the control group received 0.9% NaCl. Results showed that a decreased tumour volume, tumour growth inhibition and prolonged OS of the mice were observed in the [^177^Lu] Lu‐PSMA‐I&T treatment group via both single and fractionated doses. The potential molecular mechanism underlying the results might be that the ^177^Lu‐labelled PSMA drug increased the apoptotic activity of TNBC cells and associated endothelial cells [175]. These findings suggest that PSMA is a promising target for endogenous radioligands for the diagnosis and treatment of TNBC.

In some cases, PSMA‐targeting radiopharmaceuticals have been applied in real‐world settings. In a TNBC patient with brain metastasis, ^68^Ga‐PSMA‐11 PET/CT vividly revealed brain lesions with intense PSMA receptor activity, which was missed by traditional ^18^F‐FDG PET/CT [177]. Another pilot clinical study assessed the therapeutic effectiveness of [^177^Lu]Lu‐PSMA in an advanced TNBC patient who developed rapid progression after a wide range of systemic therapies [172]. Post‐therapeutic scintigraphy revealed intense uptake of [^177^Lu]Lu‐PSMA in the targeted lesions, indicating its feasibility for diagnosis. However, severe disease progression after two cycles of [^177^Lu]Lu‐PSMA treatment was observed in the patient, who discontinued radiotherapy despite good tolerability [172].

In summary, PSMA has potential as a vascular target for RT and TRT in the clinical diagnosis and treatment of TNBC. Nevertheless, owing to the specific localisation of PSMA in the tumour‐associated vascular endothelium, imaging or expression thresholds may not be ideal for PSMA‐targeting radiopharmaceuticals, considering such indirect targeting. However, the cross‐fire effects of TRT may compensate for this indirect targeting, resulting in promising antitumour efficacy. The specific clinical scenarios for administration and the methods to enhance efficacy remain issues that warrant consideration.

## 3 Advances in Radionuclide Delivery Systems

### 3.1 Nanoparticle‐Based Delivery Strategies

Delivery systems based on nanoparticles (NPs, 1–100 nm in diameter) have been frequently combined with RT to develop therapeutics. The specific advantages of NPs for drug delivery include biocompatibility, low toxicity, high stability, excellent penetration ability and efficient tissue retention. Moreover, NPs can be generated from any solid or liquid material, such as albumin, metals, lipids and polymers, providing a wide array of options for optimising drug delivery in RT [150, 151, 152].

Currently, several radiopharmaceutical agents have been developed using NP‐based delivery systems [91, 164]. Gold NPs (AuNPs) were functionalised with PEG chains derivatised with DOTA chelators for conjugation with ^177^Lu particles and with PEG chains linked to panitumumab for the treatment of EGFR‐expressing TNBC. The compound demonstrated strong radionuclide therapeutic effects on EGFR‐expressing TNBC [150]. Another nanodrug, ^177^Lu‐PLGA‐PTX‐Lys1‐Lys3(DOTA)‐BBN(1–14), consisted of PLGA nanoparticles radiolabelled with lutetium‐177 and further modified with the cytotoxic drug PTX and the compound Lys1‐Lys3‐DOTA‐BBN(1–14) [151]. This complex achieved targeted controlled release in TNBC cells, allowing for radiotherapy and chemotherapy in the meantime. Using a consistent principle, a different NP‐based delivery system based on a polyamidoamine (PAMAM) dendrimer functionalised with PTX and Lys1‐Lys3‐DOTA‐BBN(1–14), PAMAM‐PTX‐p‐SCN‐DOTA‐Lys1‐Lys3‐DOTA‐BBN(1–14), was introduced. Following radiolabelling with lutetium‐177, the compound revealed a selective and synergistic radiochemotherapeutic effect and excellent tumour visualisation in T47D BC cells [168].

By utilising NP‐based targeted delivery mechanisms, RT and TRT not only improve therapeutic outcomes but also reduce toxic effects, making them valuable strategies for the management of BC.

### 3.2 ADC‐Based Delivery Strategies

As we previously mentioned, the structure of an ADC is analogous to that of a TRT agent. Typically, ADCs are composed of three key components: a mAb, a linker and a cytotoxic payload [178, 179]. Such targeted drugs are designed to deliver cytotoxic payloads to specific cancer cells expressing distinct targets. Following the process of internalisation, the payload—a type of small‐molecule cytotoxic chemotherapeutic—is released, allowing accurate elimination of target‐expressing cancer cells. The mAb component plays a prominent role in the strict targeting of specific antigens expressed on the surface of cancer cells while minimising binding to normal cells [51]. Logically, the mAbs applied in ADCs can be labelled with radionuclides designed for diagnostic imaging and/or therapy.

The HER2‐targeted mAb trastuzumab has been modified with distinct radionuclides for diagnosis and treatment in the field of BC [84, 85, 86, 87, 88, 180]. ^89^Zr‐trastuzumab has been reported to determine the HER2 status across different tumour lesions in BC patients with an unclear HER2 status after standard imaging scans and biopsy [181]. PET imaging supported clinical decisions in 75% of BC cases, which indicated the feasibility of ^89^Zr‐trastuzumab imaging in the selection of personalised therapy for HER2+ BC patients [181]. In addition, trastuzumab has been conjugated with a wide array of radionuclides, such as ^131^I, ^177^Lu, ^188^Re and ^227^Th, for anticancer efficacy. For instance, ^177^Lu‐trastuzumab was modified by conjugating trastuzumab to a bifunctional chelator of DOTA and further optimising it via ^177^Lu radiolabelling [87, 88]. This compound has demonstrated high affinity for the HER2 protein, as indicated by SPECT/CT images, with particular uptake in HER2+ primary and metastatic breast tumour lesions but no localisation in HER2− BC patients. Recently, the integration of ADC‐ and NP‐based delivery approaches has further increased the accumulation of radionuclides in tumour tissues while minimising exposure to normal cells. On the basis of a HER2 nanobody called SNA004, a site‐specific coupled radiotracer of ^68^Ga‐NODAGA‐SNA004‐GSC was developed to monitor the tumour response to trastuzumab and HER2‐targeting ADCs [182]. This compound showed visual specificity and favourable sensitivity for detecting HER2 expression levels before, during and after trastuzumab and ADC treatment.

In conclusion, the ADC‐based delivery approach not only facilitates precise radiation delivery but also enhances antitumour effectiveness through the combination of targeted therapy and radiotherapy. Furthermore, the development of novel ADCs is being driven by advances in biomarker identification and characterisation. The ability to classify BC subtypes on the basis of specific biomarkers allows the design of more effective ADCs that can target the unique characteristics of individual tumours.

## 4 Challenges and Future Perspectives

### 4.1 Current Challenges

RT and TRT are expected to have good targeting and antitumour effects in the treatment of BC. To maximise the therapeutic efficacy of RT and TRT, three interrelated core challenges must be addressed: superior tumour targeting, sufficient tumour retention and rapid clearance from blood and normal tissues [49, 56, 183]. These factors collectively determine the therapeutic window—the critical balance between maximising efficacy and minimising toxicity.

Superior tumour targeting relies on the potent binding of targeted radioligands to highly specific and overexpressed biomarkers (such as receptors, antigens and enzymes) on tumour cell surfaces or within the TME. This constitutes the foundation for effective treatment, dictating the amount of radionuclide delivered to the tumour site. Furthermore, BC is a highly heterogeneous malignancy, especially for primary and metastatic lesions [184, 185]. The ER, PR and HER2 status of primary breast tumours can be altered when they develop recurrence or metastases, generally changing into tumours with increased aggressiveness and decreased expression of receptors [54, 184, 185, 186, 187]. This situation highlights the importance of identifying suitable targets to achieve effective tumour targeting. Currently, several biomarkers, including HER2, PD‐1 and EGFR, are under investigation in the field of BC. Nevertheless, most specific biomarker‐targeted radiopharmaceuticals are in the preclinical exploratory stage and lack clinical data. Potential remains for the implementation and progress of clinical research, as well as the exploration of novel tumour markers.

Sufficient tumour retention necessitates that radionuclides reaching the tumour site remain within the tumour tissue long enough for their emitted radiation (primarily α‐ or β‐particles) to exert adequate cytotoxic effects on cancer cells. Of note is the antitumour treatment of patients with BC and brain metastases. Owing to the structure of the BBB, it is difficult for a wide range of antitumour drugs to achieve effective tumour retention in metastatic brain lesions, limiting their pharmaceutical role. This applies to RT and TRT agents to some extent and necessitates improvements in pharmaceutical design. Through the application of nanoparticle‐based delivery systems or α‐particle emitters with higher linear energy transfer, RT and TRT agents may yet achieve specific efficacy in BC patients with brain metastases [188]. Moreover, conventional bifunctional conjugates typically rely on reversible ligand–target binding. This reversibility often results in insufficient radionuclide retention, significantly compromising antitumour efficacy. A recent study reported the development of covalent‐binding ligands, enabling irreversible target engagement and sufficient tumour retention in prostate cancer [49]. Analogous advances are anticipated for BC therapy in the future.

The safety profile is also crucial, in addition to therapeutic efficacy. The exposure of blood and normal organs, particularly radiosensitive organs such as the bone marrow, kidneys, salivary glands and liver, to circulating radionuclides is the primary cause of dose‐limiting toxicity [189, 190]. To improve drug tolerability, unbound radiopharmaceuticals must undergo rapid clearance from the blood circulation. Nevertheless, a fundamental dilemma exists in balancing therapeutic efficacy against safety concerns. Enhancing tumour targeting typically requires sufficient circulation time to allow the radionuclides to distribute and locate the tumour target. However, prolonged blood retention significantly increases the radiation dose to normal tissues, increasing the risk of adverse events. Certain approaches may offer potential solutions to this challenge. First, a pretargeting strategy can significantly improve pharmacokinetics. This approach decouples tumour targeting from radionuclide delivery into two distinct steps. First, a non‐radioactive targeting moiety that accumulates at the tumour site is administered, followed by the injection of a small‐molecule radionuclide that can be rapidly cleared, allowing for in situ combination via a specific chemical reaction. Second, stable linkers and chelators could be selected to minimise off‐target effects by preventing premature radionuclide dissociation before the target is reached. Third, pharmacokinetic engineering, which involves the modulation of molecular weight, charge and hydrophobicity, could enable the optimisation of blood clearance rates [38].

In conclusion, the success of RT and TRT represents the precise orchestration of tumour targeting, tumour retention and blood clearance, which is pivotal for enhancing therapeutic efficacy, widening the therapeutic window and ultimately benefiting a greater number of patients. Additionally, other factors, including economic cost together with legislation regulation of RT and TRT, also play a role in the development of such therapies.

### 4.2 Combination With Other Treatment Modalities

At present, some types of RT and TRT have achieved satisfactory outcomes in the control of BC. However, the application of RT and TRT is still worthy of consideration for patients with a heavy tumour burden and resistance to conventional treatment. In such cases, the combination of RT with other treatment modalities may improve antitumour effects and overcome drug resistance, representing a potential approach for the management of BC. Previous studies have shown that RT and TRT can be combined with diverse treatment modalities, including chemotherapy, ET, ICIs and anti‐HER2 targeted therapy [92, 106, 136, 164, 191]. Such combinations probably contribute to synergistic effects, involving a potential mechanism of enhancing the drug sensitivity of tumour cells [136, 192].

Chemotherapy remains a cornerstone in the treatment of BC, especially in aggressive subtypes. Chemotherapeutic drugs can be categorised as cell cycle–specific agents that target cells in different phases of the cell cycle and cell cycle–non‐specific agents [193, 194, 195]. For example, alkylating agents and platinum‐based derivatives are considered cell cycle non‐specific since they remain active in the resting phase of the cell [193]. They induce tumour growth suppression by facilitating the formation of cross‐links within the DNA double helix. The difference is that alkylating agents achieve this effect through alkylation reactions, with the covalent binding of alkyl groups to DNA. In contrast, plant alkaloids such as taxanes display cytotoxic effects through the prevention of microtubule disassembly during the G2/M phase of the cell cycle, which represents a well‐established cancer treatment option for solid tumours [195]. These chemotherapeutic agents can induce DNA damage, which can normally be repaired in most cases. This condition can also be observed in response to radiotherapy. Nevertheless, it would be more difficult to repair with the integration of radiotherapy and chemotherapy. For instance, the DNA crosslinking caused by alkylating agents and platinum derivatives increases the difficulty of repairing radiation‐induced single‐strand breaks in DNA. More importantly, such a combination strategy is capable of enhancing the radiosensitivity of tumour cells. During cell arrest induced by agents such as taxanes, endogenous radioprotective molecules are expressed at the lowest level, resulting in more radiosensitive tumour cells [196, 197, 198]. Logically, similar interactions can be observed for RT and TRT. A preclinical study revealed that compared with ^177^Lu‐anti‐EGFR alone, ^177^Lu‐anti‐EGFR plus docetaxel plus doxorubicin robustly inhibited the tumour growth of MDA‐MB‐231 and HCI‐002 BC xenografts [199]. Moreover, some novel TRT agents containing both chemotherapeutic agents and radionuclides, such as [^177^Lu]Lu‐BN‐PLGA(PTX), have been shown to have synergistic effects in preclinical models of BC [164].

The development of novel combination strategies is further supported by the identification of specific pathways involved in BC progression. For example, the PAM signalling pathway is frequently altered in BC, which is related to drug resistance to ET and CDK4/6 inhibitors [200, 201, 202, 203], representing a druggable target for various therapeutic agents [64, 204]. These findings indicate that specific inhibitors that target this pathway, combined with RT or TRT, may enhance therapeutic efficacy in a particular patient population.

## 5 Conclusion

BC is a malignant tumour that is highly heterogeneous in time and space. This heterogeneity may lead to differential responses to anticancer therapies, underscoring the need for personalised treatment strategies that consider the specific marker expression profile of each tumour. In this context, RT and TRT are promising therapeutic approaches that require suitable biomarkers for the development of effective agents as a fundamentally essential prerequisite. Receptor markers of HER2, ER and PR and other markers, such as Trop2, PD‐1, EGFR, GRPR and PSMA, have the potential to be used to develop RT and TRT agents for the treatment of BC.

## Author Contributions

**Yujing Tan:** conceptualisation (equal), data curation (lead), visualisation (lead), writing – original draft (lead). **Cheng Zeng:** data curation (equal), writing – original draft (equal). **Jiani Wang:** conceptualisation (equal), supervision (lead), writing – review and editing (equal). **Fei Ma:** conceptualisation (lead), supervision (lead), writing – review and editing (lead). All the authors have read, critically revised and approved the final manuscript.

## Ethics Statement

The authors have nothing to report.

## Consent

The authors have nothing to report.

## Conflicts of Interest

F.M. is a member of the *Cancer Innovation* Editorial Board. To minimise bias, he was excluded from all editorial decision‐making related to the acceptance of this article for publication. The remaining authors declare no conflicts of interest.

## Data Availability

The data that support the findings of this study are available from the corresponding author upon reasonable request.
